# Effects of pelvic endometriosis and adenomyosis on ciliary beat frequency and muscular contractions in the human fallopian tube

**DOI:** 10.1186/s12958-018-0361-y

**Published:** 2018-05-12

**Authors:** Wei Xia, Duo Zhang, Jing Ouyang, Yan Liang, Huiyu Zhang, Zhen Huang, Guiling Liang, Qian Zhu, Xiaoming Guan, Jian Zhang

**Affiliations:** 1 Department of Obstetrics and Gynaecology, International Peace Maternity and Child Health Hospital, School of Medicine, Shanghai Jiaotong University, Shanghai, China; 2 Institute of Embryo-Fetal Original Adult Disease Affiliated to School of Medicine, Shanghai Jiaotong University, Shanghai, China; 3 Department of Obstetrics & Gynaecology, Baylor College of Medicine, Houston, TX USA

**Keywords:** Endometriosis, Adenomyosis, Ciliary beat frequency (CBF), Muscular contraction, Percentage of ciliated cells

## Abstract

**Background:**

Pelvic endometriosis (EM) and adenomyosis (AM) have different effects on the fallopian tube. This study aimed to assess the transport capability of the fallopian tube in women with pelvic EM or AM.

**Methods:**

Twenty women with uterine leiomyoma (control group), 20 with adenomyosis without pelvic EM (AM group) and 35 with pelvic EM without AM (EM group) were included. EM cases were further divided into the tubal EM and non-tubal EM subgroups. Ciliary beat frequency (CBF), percentage of ciliated cells, and smooth muscle contraction were measured.

**Results:**

CBFs of the ampulla in EM cases were significantly lower than those of control and AM cases; CBFs of the ampulla and isthmus in tubal EM cases were significantly lower than those of the control group and non-tubal EM subgroup. In both the ampulla and isthmus segment, percentages of ciliated cells in EM patients were significantly lower than those of AM and control patients; the tubal EM subgroup showed significantly lower values than the control group and non-tubal EM subgroup. Amplitude-to-weight ratios of longitudinal muscular contractility in EM cases were significantly lower than control values; tubal EM cases showed significantly lower values than controls and the non-tubal EM subgroup. Contraction frequencies in EM cases were significantly lower than those of control and AM cases, in both longitudinal and circular muscles; tubal EM cases showed significantly lower values than controls and the non-tubal EM subgroup.

**Conclusion:**

EM with tubal EM damaged transport function of the fallopian tube, to varying degrees, whereas tubal function in EM without tubal EM and in AM is not altered.

## Background

Although the association of endometriosis (EM) with infertility is well recognized, the mechanisms that underlie this enigmatic disease remain unclear [1]. The overall prevalence of EM in infertile women in population-based studies varies from 20 to 50% [2]. It is generally admitted that infertility related to endometriosis can result from many factors affecting the reproductive process, including altered folliculogenesis or ovulation, defects in luteal phase function, mechanical distortion of female pelvic anatomy, impaired oocyte release or pickup, altered sperm function, and disturbances in uterine contractility [1–3]. Distortion of pelvic anatomy adversely affects sperm motility, egg transport, fertilization, and embryo transport.

Each human fallopian tube can be divided into the interstitial section, isthmus, ampulla, and fimbria. In a cross-section, the fallopian tube comprises four layers, including the serosa, subserosa, lamina propria, and innermost mucosa rich in cilia. In the reproductive process, the fallopian tube plays a crucial role in gamete transport, fertilization, and early embryo development. Gamete transportation is affected by contractions of the tubal musculature, ciliary activity, and the flow of tubal secretions [4, 5]. Unlike the ovary and uterus, which have been extensively studied and relatively well understood, the oviduct is less defined for its contribution to the reproduction [6].

Recent findings have suggested new pathomechanisms of endometriosis and adenomyosis, as both result from the same physiological mechanism of “tissue injury and repair” (TIAR) [7] and can be regarded as variants of the same disease—a dislocation of basal endometrium [8]. In EM, the ectopic endometrium (including the glands and stroma) appearing outside the uterine cavity, most often affects the ovary, pelvic peritoneum, sacral ligaments, and fallopian tubes. Meanwhile, adenomyosis is considered a benign invasion of the endometrium into the myometrium.

It is increasingly evident that the mechanism of tubal transport is much more complex than originally thought and can be affected by a wide range of factors; however, the presence of lesions that may entrap the tubes in infertile women with moderate and severe EM is clearly understood [9]. To the best of our knowledge, few studies have assessed the association of fallopian tube function in pelvic EM without tubal lesions or AM with subfertility. Kissler and coworkers examined sperm utero-tubal transport by hysterosalpingoscintigraphy (HSSG) and revealed that sperm transport is significantly reduced in infertile women with AM combined with pelvic EM, with pregnancy rate diminished due to this impairment [10, 11]. Studies analyzing the effects of peritoneal fluid samples from women with endometriosis on ciliary beat frequency (CBF) concluded that CBF is significantly lower in women with endometriosis compared with fertile controls [12, 13]. Reeve and coworkers demonstrated that sperm binding to the tubal epithelium may be disrupted in women with endometriosis [14].

Currently, it is commonly admitted that the function of the fallopian tube can be precisely assessed by CBF and tubal smooth muscle contraction. However, no study simultaneously evaluating the transport capacity of cilia and muscles of the fallopian tube by the above methods in women with pelvic EM and AM is available. The present study aimed to assess the transport capability of the fallopian tube by CBF and tubal smooth muscle contraction in women with pelvic EM or AM.

## Methods

### Participants

This study was approved by the institutional ethics committee of the International Peace Maternity and Child Health Hospital in Shanghai, China (No. GKLW 2016-42). Written informed consent was obtained from each participant before enrolment.

Fallopian tube specimens were obtained were obtained between June 2016 and August 2017 at the Department of Obstetrics and Gynecology of the International Peace Maternity and Child Health Hospital, Shanghai. The specimens were obtained from 20 women with uterine leiomyoma (control group), 20 with adenomyosis without pelvic EM, and 35 with pelvic EM without AM according to the MRI before operation (all EM patients had ovarian endometrioma). All the patients with uterine leiomyoma and adenomyosis were treated with hysterectomy and salpingectomy. All the diagnosis were depended on the pathological examinations. All women had a regular menstrual cycle, and had not used hormonal medication within 3 months of surgery; they had no history of tubal surgery. As shown by pathological examination, all the women included in the present study were in the proliferative stage.

Before surgery, the participants were interviewed personally using a standard questionnaire on paper the day before operation for sociodemographic characteristics, reproductive history, the visual analogue scale (VAS) score, and the Mansfield-Voad-Jorgensen (MVJ) score, which ranging from 1 (spotting) to 6 (gushing) to assess the menstrual blood loss. An MVJ score equal to or > 5 is considered hypermenorrhoea [15].

### Collection of human fallopian tubes

The ampulla and isthmus of the fallopian tube in each participant were placed in 50 mL tubes containing the culture medium (Dulbecco modified Eagle medium) and we transported the specimens from operation room to the lab for experiments in 10 min. The fallopian tubes were rinsed several times to remove all visible blood, and the muscular and epithelial tissues were dissected.

### Hematoxylin and eosin (H&E) staining and immunohistochemistry

Specimens were fixed with paraformaldehyde for 48 h at 4 °C, dehydrated in graded ethanol, paraffin embedded, and sliced into 5-μm-thick sections using a microtome (Leica, Wetzlar, Germany). Subsequently, the sections were deparaffinized with xylene and stained with H&E. For immunohistochemistry, the sections were treated with 3% hydrogen peroxide. After three washes in phosphate-buffered saline (PBS, Goodbio technology Co., Ltd., Wuhan, China), 10% normal mouse serum was applied for 15 min, followed by incubation with mouse monoclonal anti-CD10 (clone 56C6, Thermo Scientific) antibody overnight in a humidified chamber at 4 °C. Then, the sections were incubated with goat anti-mouse secondary antibody for 30 min at room temperature, sand detected by 3,30-diaminobenzidine tetra-hydrochloride solution (DAB, Goodbio technology Co., Ltd., Wuhan, China) [16].

### Diagnostic criteria of tubal endometriosis

Tubal EM diagnosis was confirmed by final postoperative pathology based on H&E staining and CD10 immunohistochemistry. Tubal EM cases were evaluated and classified as proposed by Kurman and coworkers [17]. All assessments were performed independently by two experienced pathologists, in a blinded manner. Any disagreements between the two pathologists required evaluation by a third pathologist, and the final results were determined by the two pathologists.

### Measurement of CBF

Small (1–2 mm^2^) pieces of oviduct epithelium were separated from the isthmus-ampulla portion of the fallopian tube. CBF was measured on an inverted bright-field microscope (Leica DMi8, Leica Co., Germany) equipped with a × 40 objective. Moving cilia were imaged and analyzed as previously described [18]*.* Five regions were randomly selected in each specimen, and two specimens per patient were measured. Thus, a total of 10 CBF measurements were performed per sample and averaged.

### Percentage of ciliated cells

Specimens were fixed in paraformaldehyde solution for hematoxylin and eosin (H&E) staining for 48 h at 4 °C, dehydrated in a graded ethanol series, and paraffin embedded. Sections (5 μm) were cut on a microtome (Leica, Wetzlar, Germany). The morphology of the oviduct epithelium was analyzed under a microscope (DSM 2500; Leica Stereozoom, Leica Microsystems, Heerbrugg, Switzerland). Five images were randomly acquired per sample. In each image, all cells as well as ciliated cells were counted (× 400 magnification) [19, 20].

### Contraction of muscle strips

The isthmus-ampulla segments of fallopian tube samples were immediately removed and placed in a glass beaker containing cooled Krebs solution. Circular and longitudinal muscle strips (2 mm × 8 mm) of human oviducts were prepared. The isolated smooth muscle strips were placed in isolated organ baths filled with Krebs solution (5.9 mM KCl, 1.2 mM NaH_2_PO_4_, 1.2 mM MgCl_2_, 120.6 mM NaCl, 15.4 mM NaHCO_3_, 11.5 mM glucose and 2.5 mM CaCl_2_), incubated in an environment containing 95% O_2_ and 5% CO_2_, and connected with tension transducers. The strips were equilibrated for 30 min, and stretched with a tension of 0.3 g.

### Statistical analysis

Statistical analyses were performed with GraphPad Prism 6.0 (GraphPad Soft-ware Inc., California, USA). Data are mean ± SEM, and were compared by unpaired Student’s t-test. *P* < 0.05 was considered statistically significant.

## Results

Of the 35 women with pelvic endometriosis, 11 and 24 were laparoscopically diagnosed with endometriosis stage III and endometriosis stage IV, respectively, according to the revised American Society for Reproductive Medicine (ASRM) classification (ASRM, 1997).

Table 1 shows the characteristics of the recruited patients. No differences were obtained between control patients and the AM or EM group, in age, gravidity, parity, and baseline MVJ score. Compared with control values, VAS scores in the AM (*p* < 0.001) and EM (*p* < 0.001) groups were significantly higher.

**Table 1:** Characteristics of the recruited patients

Variables	Control group (*n* = 20)	AM group (*n* = 20)	EM group (*n* = 35)
Age^a^ (year)	47.2 ± 4.8	44.4 ± 5.2	43.4 ± 5.1
BMI^a^ (kg/m^2^)	23.8 ± 2.2	21.0 ± 1.9	21.8 ± 2.3
Gravidity(n)	1.9 ± 1.4	1.8 ± 1.5	1.25 ± 0.9
Parity (n)	1.1 ± 0.9	0.9 ± 0.7	1.0 ± 0.5
Baseline of dysmenorrhea VAS score^a^	1.7 ± 1.6^c^	5.9 ± 2.7	5.7 ± 2.6
Baseline of MVJ score^b^	4.8 ± 0.9	4.7 ± 0.9	4.2 ± 1.0

Data are mean ± standard deviation (SD)

*AM* adenomyosis, *EM* endometriosis

^a^The degree of baseline pain was evaluated by the pre-surgical visual analogue scale (VAS) score (0–10 indicates no pain to severe pain)

^b^Baseline menstrual blood loss was assessed using the pre-surgical Mansfield-Voda-Jorgensen (MVJ) score, ranging from 1 (spotting) to 6 (gushing)

^c^Significant difference compared with the AM and EM groups (both *p* < 0.001)

According to pathological findings, 24 of the 35 women in the EM group had lesions involving the fallopian tubes, and were included in the tubal EM subgroup; the remaining 11 were included in the non-tubal EM subgroup.

**Fig. 1:**
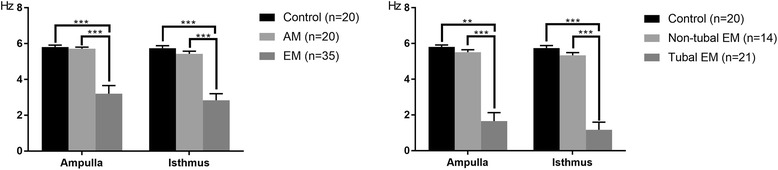
Ciliary beat frequency (CBF). Data are mean ± SEM. **p < 0.01, ***p < 0.001

The CBF of the ampulla in the EM group (3.202 ± 0.077 Hz) was significantly lower than those of the control (5.811 ± 0.021 Hz) and AM (5.725 ± 0.018 Hz) groups (both *p* < 0.001). The CBF of the isthmus segment in the EM group (2.842 ± 0.061 Hz) was also significantly lower than those of the control (5.744 ± 0.031 Hz) and AM (5.432 ± 0.034 Hz) groups (both *p* < 0.001; Fig. 1). There were no significant differences in CBF between the control and AM groups, or between the control group and non-tubal EM subgroup. CBFs of the ampulla and isthmus in the tubal EM subgroup (1.661 ± 0.102 Hz and 1.173 ± 0.093 Hz, respectively) were both significantly lower than those of the control group and non-tubal EM subgroup (5.510 ± 0.037 Hz and 5.339 ± 0.041 Hz, respectively).

**Fig. 2:**
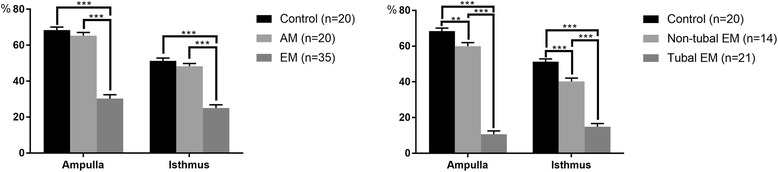
Percentage of ciliated cells. Data are mean ± SEM. **p < 0.01, ***p < 0.001

**Fig. 3:**
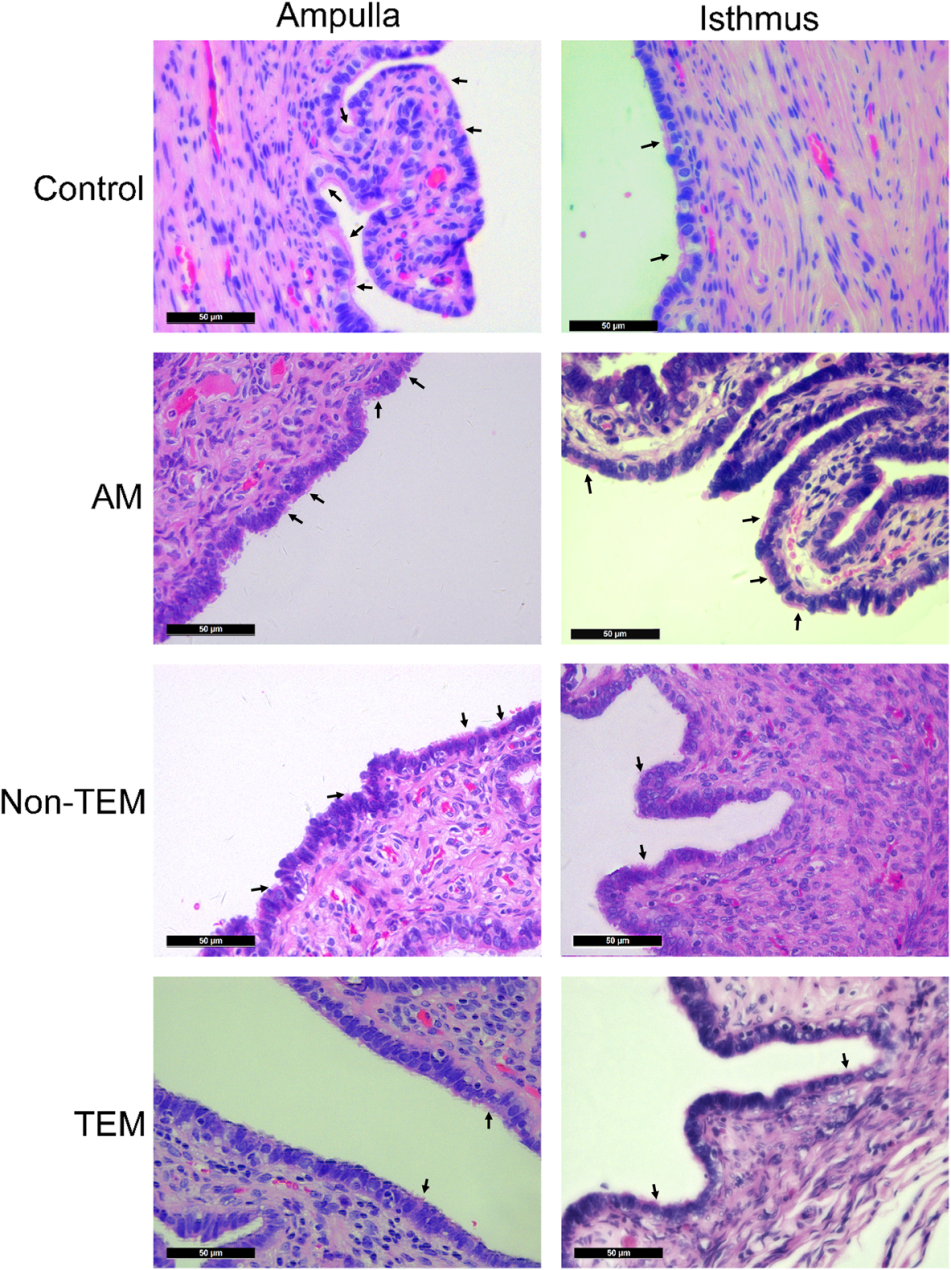
Histological analysis of human fallopian tubes. Light microscopy images showing that the non-tubal EM (tubal endometriosis) and tubal EM subgroups had less ciliated cells. Arrows indicate ciliated cells

Figures 2 and 3 shows the percentages of ciliated cells in ampulla and isthmus samples from the fallopian tubes. In both the ampulla and isthmus, the percentages of ciliated cells in the EM group (30.319 ± 0.359% and 25.601 ± 0.305%, respectively) were significantly lower (both *p* < 0.001) than those of the AM (65.312 ± 0.408% and 48.314 ± 0.339%, respectively) and control (68.339 ± 0.381% and 51.281 ± 0.351%, respectively) groups; the values in the tubal EM subgroup (10.561 ± 0.414% and 15.849 ± 0.407%, respectively) were both significantly lower (both *p* < 0.001) than those of the control group and non-tubal EM subgroup (59.961 ± 0.539% and 40.227 ± 0.501%, respectively); the values in the non-tubal EM subgroup were both significantly lower (*p* < 0.01 and *p* < 0.001, respectively) than those of the control group. There were no significant differences between control cases and the AM group.

**Fig. 4:**
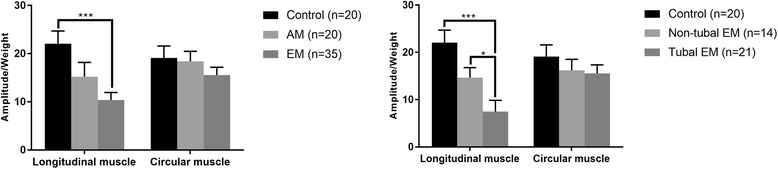
Amplitude/Weight ratio of the tubal smooth muscle spontaneous contraction. Data are mean ± SEM. *p < 0.05, ***p < 0.001

Figure 4 summarize the spontaneous contractile activities of the isthmus-ampulla segments of fallopian tube strips. The amplitude/weight ratio of longitudinal muscular contractility in the EM group (10.357 ± 0.269) was significantly (*p* < 0.001) lower than control values (22.061 ± 0.594). The tubal EM subgroup (7.491 ± 0.496) showed significantly lower values than the control group and non-tubal EM subgroup (14.657 ± 0.569) (*p* < 0.001 and *p* < 0.05, respectively). There were no significant differences in circular muscle contractility among groups.

**Fig. 5:**
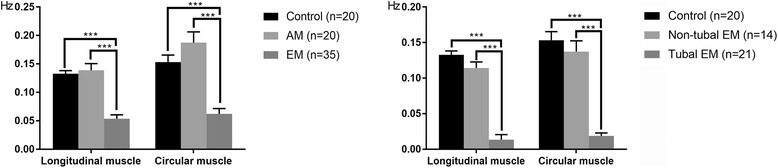
Frequency of the tubal smooth muscle spontaneous contraction. Data are mean ± SEM. **p < 0.01, ***p < 0.001

Figure 5 shows the frequency of contraction in the EM group was significantly (*p* < 0.001) lower than those of the control and AM groups, both in the longitudinal (0.054 ± 0.002 Hz, 0.133 ± 0.001 Hz, and 0.13 ± 0.003 Hz, respectively) and circular (0.062 ± 0.003 Hz, 0.153 ± 0.003 Hz, and 0.187 ± 0.004 Hz, respectively) muscles. Frequencies of contraction in the tubal EM subgroup were significantly (*p* < 0.001) lower than those of the control group and non-tubal EM subgroup, both in longitudinal (0.013 ± 0.002 Hz, 0.133 ± 0.001 Hz, and 0.114 ± 0.002 Hz, respectively) and circular (0.019 ± 0.001 Hz, 0.153 ± 0.003 Hz, 0.127 ± 0.004 Hz, respectively) muscles.

## Discussion

To the best of our knowledge, this is the first report assessing the effects of pelvic endometriosis, tubal endometriosis, and adenomyosis on ciliary beat frequency and contraction of tubal smooth muscle in the human fallopian tube. AM and EM without tubal EM did not affect tubal function. EM with tubal EM resulted in lower ciliary beat frequency, lower ciliated cells percentage, weaker muscular contractile activity, and lower contraction frequency.

Moderate to severe EM may cause pelvic adhesions and anatomical changes. The morphology and function of the fallopian tube are susceptible to damage because it is located between the uterus and ipsilateral ovary. As early as 1984, Schenken and coworkersshowed that pregnancy rates are lower among monkeys with moderate or severe endometriosis, which showed pelvic adhesion involving ovaries and tubes [21]. According to the TOP score [22] and endometriosis fertility index (EFI) [23, 24] to predict the reproductive outcomes of EM patients, the results are mostly affected by the function of tubal function.

Using objective measures to evaluate fallopian tube function, including CBF, percentage of ciliated cells, and smooth muscle contraction amplitude and frequency, we demonstrated for the first time that EM with tubal EM damaged transport function of the fallopian tube, to varying degrees, whereas tubal function in EM without -tubal EM is not altered. Tubal EM refers to functional endometrial glands and stroma implant involving the fallopian tube. Currently, only a few studies have assessed tubal EM prevalence, with results varying widely. Some of them are retrospective trials with small sample sizes or case reports [25–27]. Furthermore, there is no research evaluating associations of internal tubal environment, tubal muscle contraction, tubal cilia movement, and reproductive function with tubal EM. The tubal EM layer may infiltrate the serosa, muscle, and mucosa, and the ectopic endometrial tissue may give rise to a variety of lesions, including endometrial-type polyps, tubal adenomyosis (analogous to salpingitis isthmica nodosa), and intraluminal endometriosis, with occlusion of one or both tubal lumens. In this study, endometriosis lesions were found in the fallopian tubes of 60% (21/35) EM patients; therefore, tubal involvement in moderate and severe EM is common. Tubal inflammation, edema, damage of mucosal folds, and fibrosis hyperplasia can decrease tubal transport function in the epithelium and muscle. This study also found for the first time that AM did not affect the transport function of fallopian tubes, indicating that adenomyosis and EM differ in pathogenesis. Endometriosis lesions in AM without pelvic EM are confined to the myometrium, and the fallopian tubes are often not involved.

The abnormal local microenvironment of pelvic and tube invading by EM lesion, including immune environment, oxidative stress, high estrogen, increased local inflammatory factors, etc., can lead to cell damage and apoptosis [1]. In this study, although the CBF in non-tubal EM subgroup has not been changed, but the local cilia cells were less than the normal control group, suggesting that endometriosis, regardless of tubal involvement or not, can cause different degrees of damage of ciliated cells.

Although the importance of each of the ciliary and muscular motion mechanisms remains unclear, there is evidence that ciliary activity may play a dominant role in the transport of normal gametes and embryos. With reduced muscular activity, total transport rates through the ampulla are unchanged, suggesting that ciliary motion alone is capable of transporting ova to the site of fertilization in the absence of muscle contractility [27, 28]. Conversely, women with the ‘immotile cilia syndrome’, or Kartagener’s syndrome, may suffer from infertility because of a dyskinetic ciliary activity in the fallopian tubes [29, 30]. We evaluated the functions of the tubal cilia and smooth muscle objectively, for the first time in the related research field studying the impact of EM on transport function in fallopian tubes. Currently, it is widely admitted that fallopian tube function can be precisely assessed by ciliary beat frequency (CBF) and smooth muscle contraction. The current findings revealed that cilia and muscle functions were impaired, indicating that EM damages the tubal epithelium and muscle layers. Considering the great importance of cilia and smooth muscle to the reproductive function of the fallopian tube, our study provide a new perspective for the research of the tubal function in AM and EM.

This study had several limitations. First, because most of the patients with mild endometriosis have operations to get pregnant, we cannot do the salpingectomy. Patients with stage I and stage II EM were not included, and whether mild EM impacts fallopian tube function was not evaluated. Secondly, other tubal segments (interstitial and fimbria) were not assessed. Thirdly, we did not observe the tubal ultrastructure, e.g. by transmission electron microscopy, which detects ultrastructural defects of cilia. Fourthly, animal experiments would have helped unveil the mechanisms. Further studies assessing the impact of mild EM on tubal function and animal experiments evaluating the impact on transport function in vivo are warranted. In addition, drugs, surgery, or other means, which can reverse the functional impairment of the fallopian tubes deserve further investigation.

## Conclusion

Fallopian tubes in women with moderate and severe EM, especially EM with tubal EM, show lower ciliary beat frequency, lower ciliated cells percentage, weaker muscular contractile activity, and lower contraction frequency. These findings suggest that the fallopian tube plays an important role in EM-related infertility. AM does not affect tubal function, indicating that there are other factors responsible for AM-related infertility.

## Abbreviations

AM: Adenomyosis
CBF: Ciliary beat frequency
EM: Endometriosis
MVJ: Mansfield-Voad-Jorgensen
VAS: Visual analogue scale

## References

[1] de Ziegler D, Borghese B, Chapron C (2010). Endometriosis and infertility: pathophysiology and management. Lancet.

[2] Tanbo T, Fedorcsak P (2017). Endometriosis-associated infertility: aspects of pathophysiological mechanisms and treatment options. Acta Obstet Gynecol Scand.

[3] Holoch KJ, Lessey BA (2010). Endometriosis and infertility. Clin Obstet Gynecol.

[4] Raidt J, Werner C, Menchen T, Dougherty GW, Olbrich H, Loges NT, Schmitz R, Pennekamp P, Omran H (2015). Ciliary function and motor protein composition of human fallopian tubes. Hum Reprod.

[5] Lyons RA, Saridogan E, Djahanbakhch O (2006). The reproductive significance of human fallopian tube cilia. Hum Reprod Update.

[6] Li S, Winuthayanon W (2017). Oviduct: roles in fertilization and early embryo development. J Endocrinol.

[7] Leyendecker G, Wildt L, Mall G (2009). The pathophysiology of endometriosis and adenomyosis: tissue injury and repair. Arch Gynecol Obstet.

[8] Leyendecker G, Herbertz M, Kunz G, Mall G (2002). Endometriosis results from the dislocation of basal endometrium. Hum Reprod.

[9] Burns WN, Schenken RS (1999). Pathophysiology of endometriosis-associated infertility. Clin Obstet Gynecol.

[10] Kissler S, Hamscho N, Zangos S, Gatje R, Muller A, Rody A, Dobert N, Menzel C, Grunwald F, Siebzehnrubl E, Kaufmann M (2005). Diminished pregnancy rates in endometriosis due to impaired uterotubal transport assessed by hysterosalpingoscintigraphy. BJOG.

[11] Kissler S, Zangos S, Wiegratz I, Kohl J, Rody A, Gaetje R, Doebert N, Wildt L, Kunz G, Leyendecker G, Kaufmann M (2007). Utero-tubal sperm transport and its impairment in endometriosis and adenomyosis. Ann N Y Acad Sci.

[12] Papathanasiou A, Djahanbakhch O, Saridogan E, Lyons RA (2008). The effect of interleukin-6 on ciliary beat frequency in the human fallopian tube. Fertil Steril.

[13] Lyons RA, Djahanbakhch O, Saridogan E, Naftalin AA, Mahmood T, Weekes A, Chenoy R (2002). Peritoneal fluid, endometriosis, and ciliary beat frequency in the human fallopian tube. Lancet.

[14] Reeve L, Lashen H, Pacey AA (2005). Endometriosis affects sperm-endosalpingeal interactions. Hum Reprod.

[15] Mansfield PK, Voda A, Allison G (2004). Validating a pencil-and-paper measure of perimenopausal menstrual blood loss. Womens Health Issues.

[16] Groisman GM, Meir A (2003). CD10 is helpful in detecting occult or inconspicuous endometrial stromal cells in cases of presumptive endometriosis. Arch Pathol Lab Med.

[17] Blaustein's Pathology of the Female Genital Tract. 2011. 10.1007/978-1-4419-0489-8.

[18] Chen JJ, Lemieux BT, Wong BJ (2016). A low-cost method of ciliary beat frequency measurement using iPhone and MATLAB: rabbit study. Otolaryngol Head Neck Surg.

[19] Chen S, Einspanier R, Schoen J (2013). In vitro mimicking of estrous cycle stages in porcine oviduct epithelium cells: estradiol and progesterone regulate differentiation, gene expression, and cellular function. Biol Reprod.

[20] Zhao W, Zhu Q, Yan M, Li C, Yuan J, Qin G, Zhang J (2015). Levonorgestrel decreases cilia beat frequency of human fallopian tubes and rat oviducts without changing morphological structure. Clin Exp Pharmacol Physiol.

[21] Schenken RS, Asch RH, Williams RF, Hodgen GD (1984). Etiology of infertility in monkeys with endometriosis: luteinized unruptured follicles, luteal phase defects, pelvic adhesions, and spontaneous abortions. Fertil Steril.

[22] Fujishita A, Khan KN, Masuzaki H, Ishimaru T (2002). Influence of pelvic endometriosis and ovarian endometrioma on fertility. Gynecol Obstet Investig.

[23] Adamson GD, Pasta DJ (2010). Endometriosis fertility index: the new, validated endometriosis staging system. Fertil Steril.

[24] Tomassetti C, Geysenbergh B, Meuleman C, Timmerman D, Fieuws S, D'Hooghe T (2013). External validation of the endometriosis fertility index (EFI) staging system for predicting non-ART pregnancy after endometriosis surgery. Hum Reprod.

[25] Jenkins S, Olive DL, Haney AF (1986). Endometriosis: pathogenetic implications of the anatomic distribution. Obstet Gynecol.

[26] Clement PB (2007). The pathology of endometriosis: a survey of the many faces of a common disease emphasizing diagnostic pitfalls and unusual and newly appreciated aspects. Adv Anat Pathol.

[27] Fortier KJ, Haney AF (1985). The pathologic spectrum of uterotubal junction obstruction. Obstet Gynecol.

[28] Halbert SA, Becker DR, Szal SE (1989). Ovum transport in the rat oviductal ampulla in the absence of muscle contractility. Biol Reprod.

[29] Abu-Musa A, Nassar A, Usta I (2008). In vitro fertilization in two patients with Kartagener's syndrome and infertility. Gynecol Obstet Investig.

[30] Halbert SA, Patton DL, Zarutskie PW, Soules MR (1997). Function and structure of cilia in the fallopian tube of an infertile woman with Kartagener's syndrome. Hum Reprod.

